# The *Vitis vinifera *sugar transporter gene family: phylogenetic overview and macroarray expression profiling

**DOI:** 10.1186/1471-2229-10-245

**Published:** 2010-11-12

**Authors:** Damien Afoufa-Bastien, Anna Medici, Julien Jeauffre, Pierre Coutos-Thévenot, Rémi Lemoine, Rossitza Atanassova, Maryse Laloi

**Affiliations:** 1 UMR-CNRS-UP 6503 - LACCO - Laboratoire de Catalyse en Chimie Organique - Equipe Physiologie Moléculaire du Transport de Sucres - Université de Poitiers - Bâtiment Botanique - 40 Avenue du Recteur Pineau, 86022 Poitiers cedex, France; 2 UMR Génétique et Horticulture (GenHort) - INRA/INH/UA - BP 60057 - 49071 Beaucouzé cedex, France

## Abstract

**Background:**

In higher plants, sugars are not only nutrients but also important signal molecules. They are distributed through the plant *via *sugar transporters, which are involved not only in sugar long-distance transport *via *the loading and the unloading of the conducting complex, but also in sugar allocation into source and sink cells. The availability of the recently released grapevine genome sequence offers the opportunity to identify sucrose and monosaccharide transporter gene families in a woody species and to compare them with those of the herbaceous *Arabidopsis thaliana *using a phylogenetic analysis.

**Results:**

In grapevine, one of the most economically important fruit crop in the world, it appeared that sucrose and monosaccharide transporter genes are present in 4 and 59 loci, respectively and that the monosaccharide transporter family can be divided into 7 subfamilies. Phylogenetic analysis of protein sequences has indicated that orthologs exist between *Vitis *and *Arabidospis*. A search for *cis*-regulatory elements in the promoter sequences of the most characterized transporter gene families (sucrose, hexoses and polyols transporters), has revealed that some of them might probably be regulated by sugars. To profile several genes simultaneously, we created a macroarray bearing cDNA fragments specific to 20 sugar transporter genes. This macroarray analysis has revealed that two hexose (*VvHT1*, *VvHT3*), one polyol (*VvPMT5*) and one sucrose (*VvSUC27*) transporter genes, are highly expressed in most vegetative organs. The expression of one hexose transporter (*VvHT2*) and two tonoplastic monosaccharide transporter (*VvTMT1*, *VvTMT2*) genes are regulated during berry development. Finally, three putative hexose transporter genes show a preferential organ specificity being highly expressed in seeds (*VvHT3*, *VvHT5*), in roots (*VvHT2*) or in mature leaves (*VvHT5*).

**Conclusions:**

This study provides an exhaustive survey of sugar transporter genes in *Vitis vinifera *and revealed that sugar transporter gene families in this woody plant are strongly comparable to those of herbaceous species. Dedicated macroarrays have provided a *Vitis *sugar transporter genes expression profiling, which will likely contribute to understand their physiological functions in plant and berry development. The present results might also have a significant impact on our knowledge on plant sugar transporters.

## Background

In plants, sugars (sucrose, monosaccharides, polyols) are important molecules that constitute not only metabolites but also nutrients, osmotic and signal molecules. In numerous species, sucrose is the most prevalent sugar produced in photosynthetic organs (source) and transported *via *the phloem over long distances to heterotrophic organs (sink), which depend on a constant supply of carbohydrates [1]. In sink organs, sucrose is either directly imported or cleaved by cell wall-bound invertases into monosaccharides (glucose and fructose), that can be taken up by the sink cells [2]. In some species, sugar alcohols (polyols), such as mannitol, sorbitol and galactinol can also be transported on top of sucrose for long-distance carbon partitioning [3]. In addition to this long-distance transport, sugars can also be allocated in the different organelles of source and sink cells, and more and more biochemical and molecular studies argue for the transport of hexoses into the chloroplast [4] the vacuoles [5], and the Golgi apparatus [6]. Therefore, it is now clearly established that not only the loading and the unloading of the conducting complex, but also the allocation of sugars into source and sink cells is controlled by sugar transporters mediating the transport of sucrose [7-9], reducing monosaccharides [10], or polyols [11-13]. Since the cloning of the first monosaccharide transporter in *Chlorella *[14], the first sucrose transporter in *Spinacia oleracea *[15], and the first polyol transporter in *Apium graveolens *[11], many genes belonging to these families have been isolated from various species. The complete *Arabidopsis *genome has been described to contain 9 sucrose transporter-like sequences [8] and a monosaccharide transporter(-like) gene family, including 53 members grouped into 7 subfamilies [10]. Furthermore, the evolutionary analysis of plant monosaccharide transporters revealed that these seven subfamilies are ancient in land plants [16].

Despite the progress made in identifying genes encoding sugar transporters, little is known about the transcriptional regulation of these genes. *Arabidopsis *microarray data (Genevestigator: https://www.genevestigator.com; The BAR: http://bbc.botany.utoronto.ca) and some plant transporter gene expression patterns have indicated that developmental and environmental factors could regulate the expression of sugar transporters. Furthermore, evidence is provided that the expression of some sugar transporter genes is regulated by sugars as described for sugar transporter genes in yeast [17], for *VvHT1*, a grapevine hexose transporter [18-20] and for sucrose transporter genes from rice, *OsSUT1 *[21] and sugar beet, *BvSUT1 *[22-24]. All these data suggest that the expression of sugar transporters might be regulated at the transcriptional level by distinct but usually converging signalling pathways, depending on either developmental and environmental cues or metabolic and hormonal signals. In spite of the evidence for the role of sugar signalling in the transcriptional control of some transporter genes, the *in silico *analysis of promoter regions of different genes involved in carbon metabolism, sugar storage, mobilization and transport clearly demonstrates the absence of common sugar specific *cis*-elements [25-27]. This analysis is consistent with the fact that in plants, several types of transcription factors (bZIP, WRKY, AP2, MYB, B3, EIN3) are required for sugar signalling and are involved in sugar-regulation of gene expression [27,28]. Considering that the analysis of sugar transporter orthologs in different species might help to better understand their biological function, we analyzed the recently sequenced *Vitis vinifera *genotype PN40024 [29] in order to identify sugar transporter gene families in this species. This work will represent the first exhaustive analysis for sugar transporters in ligneous plant as most of the already known sugar transporters have been characterized from herbaceous species. In woody plants, only 4 sucrose transporters have been already described in *Vitis *[30-32] (GenBank: AF439321), 2 in *Citrus sinensis *(GenBank: AY098891, AY098894), 2 in *Hevea brasiliensis *(GenBank: ABJ51934, ABK60189) and one in *Juglans regia *[33,34]. Seven hexose transporters in *Vitis *[35-37], 2 in *Juglans regia *[34] and few polyol transporters in *Prunus cerasus *[38], in *Malus domestica *[39] and in *Olea europea *[40] were also reported. Furthermore during the last decade, *Vitis vinifera *has become an interesting model to study fruit maturation. It is now clearly established that the onset of ripening (veraison) is characterized by an important accumulation of glucose and fructose in vacuoles of the mesocarp cells [41]. In grapevine, sucrose is the main carbohydrate used for long distance transport and after reaching the phloem of the berry, it is unloaded into the apoplast, possibly cleaved by apoplastic invertases, and sucrose or hexoses can than be transported into the mesocarp. In the cytoplasm of the mesocarp cells, sucrose and hexoses must be transported into the vacuole *via *tonoplastic transporters. The identification and the characterization of sugar transporter genes in *Vitis vinifera *are therefore important steps in understanding the roles of these proteins in grapevine development as well as in grape ripening process and may further highlight our knowledge on plant sugar transporters.

The present study reports on the identification of sucrose and monosaccharide-like transporter genes in the *Vitis vinifera *genome, on their phylogenetic analysis in comparison with *Arabidopsis *transporters, on their promoter sequences analysis. The construction of specialized cDNA macroarrays used to determine the expression pattern for 20 of these genes in grapevine vegetative organs and during berry ripening is also described.

## Results

### Identification of sugar transporters from *Vitis vinifera*

**Figure 1:**
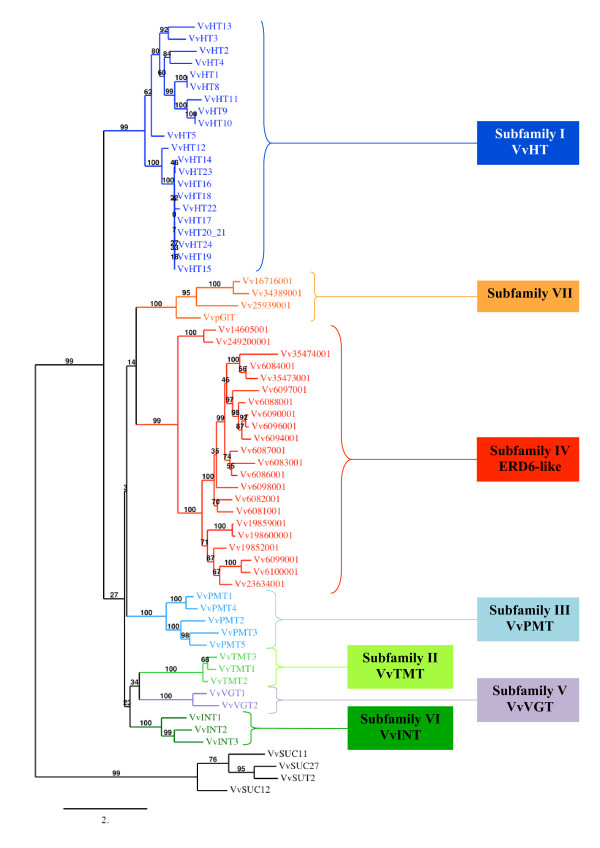
**Maximum likelihood phylogeny of *Vitis vinifera *sugar transporter proteins**. The tree was produced using MUSCLE and PhyML with the JTT amino acid substitution model, a discrete gamma model with 4 categories and an estimated shape parameter of 1.213. Bootstrapping was performed with 100 replicates. For accession numbers of *Vitis *sugar transporter sequences see Additional file 1. *Vitis *ORFs names were simplified, Vv indicating GSVIVT000.

Blastp searches of the grapevine genome proteome 8× database, using the amino acid sequences of the 9 sucrose transporters and the 53 monosaccharide transporters from *A. thaliana *as query, allowed the identification of 65 ORFs encoding putative sugar transporters in *V. vinifera *(Additional file 1). Among these ORFs, only 4 encode previously described sucrose transporters [30-32] (GenBank: AF439321) and no additional one could be identified. The 61 other ORFs seem to encode putative monosaccharide transporters (MST). Phylogenetic analysis of the 65 *V. vinifera *identified protein sequences using the maximum likelihood (ML) method (Figure 1) reveals that sucrose and monosaccharide transporters form two separate groups. Furthermore in agreement with the phylogeny observed for *A. thaliana *MST [10,16], 7 distinct subfamilies (I-VII) could be clearly identified in the *Vitis *monosaccharide transporter group (Additional file 1 and Figure 1).

### *Vitis vinifera *Sucrose Transporters (VvSUC, VvSUT)

**Figure 2:**
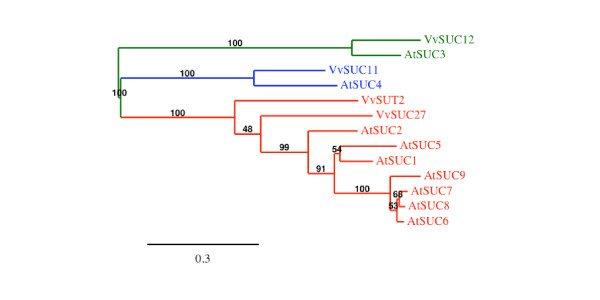
**Maximum likelihood phylogeny of *Vitis vinifera *and *Arabidopsis thaliana *sucrose transporter proteins**. The tree was produced using MUSCLE and PhyML with the JTT amino acid substitution model, a discrete gamma model with 4 categories and an estimated shape parameter of 0.872. Bootstrapping was performed with 100 replicates. Accession numbers for *Arabidopsis thaliana *transporters are: At1g71880 (AtSUC1), At1g22710 (AtSUC2), At2g02860 (AtSUC3), At1g09960 (AtSUC4), At1g71890 (AtSUC5), At5g43610 (AtSUC6), At1g66570 (AtSUC7), At2g14670 (AtSUC8), At5g06170 (AtSUC9); for *Vitis *ones see Additional file 1.

The 4 amino acids sequences encoding the already described sucrose transporters named VvSUC11/VvSUT1, VvSUC12, VvSUC27 and VvSUT2 [30-32] share 40 to 59% similarity between each other and fall into three sucrose transporter subgroups already described (Figure 2) [7-9]. VvSUT2 and VvSUC27 shows 59% similarity and belong to the dicots specific SUT1 subfamily including high affinity sucrose transporters exhibiting apparent *K_m _*value between 0.07 and 2 mM. However, VvSUC27 has been described to be a low-affinity/high-capacity sucrose transporter showing a *K_m _*value for sucrose ranged between 8.0 and 10.5 mM [42]. The structure of *VvSUT2 *and *VvSUC27 *genes is quite similar, both being around 2380 bp long and containing 4 exons separated by 3 introns (Additional file 1). VvSUC12 shows 66.6% similarity with AtSUC3 and presents an extended domain at the N terminus and an elongated central cytoplasmic loop; two structural characteristics specific to the SUT2/SUC3 subfamily [43]. Furthermore, *VvSUC12 *gene is a very long gene (more than 10 kb) containing 14 exons interrupted by 13 introns (Additional file 1); such exon/intron organization is also described for *AtSUC3*. The *K_m _*value for sucrose (1.36 mM) reported for this transporter [32] seems, however, higher than that described for other members of this subfamily, showing either a low affinity (AtSUC3: *K_m _*= 11.7 mM) or no sucrose transport function. Finally, although VvSUC11 has a high affinity for sucrose (*K_m _*= 0.88 mM; [32]) it shows 67.9% similarity with AtSUC4 and falls into the SUT4 subfamily including all low-affinity plant sucrose transporters with *K_m _*value ranging between 5 mM and 6 mM.

### *Vitis vinifera *putative Hexose Transporters (VvHT; subfamily I)

**Figure 3:**
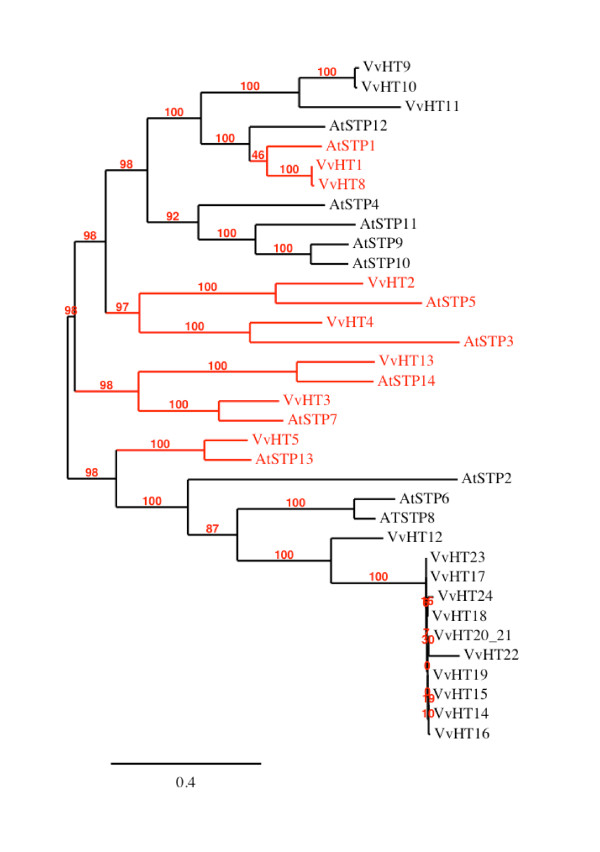
**Maximum likelihood phylogeny of *Vitis vinifera *and *Arabidopsis thaliana *hexose transporter proteins**. The tree was produced using MUSCLE and PhyML with the JTT amino acid substitution model, a discrete gamma model with 4 categories and an estimated shape parameter of 1.025. Bootstrapping was performed with 100 replicates. Accession numbers for *Arabidopsis thaliana *transporters are: At1g11260 (AtSPT1), At1g07340 (AtSTP2), At5g61520 (AtSTP3), At3g19930 (AtSTP4), At1g34580 (AtSTP5), At3g05960 (AtSTP6), At4g02050 (AtSTP7), At5g26250 (AtSTP8), At1g50310 (AtSTP9), At3g19940 (AtSTP10), At5g23270 (AtSTP11), At4g21480 (AtSTP12), At5g26340 (AtSTP13), At1g77210 (AtSTP14); for *Vitis *ones see Additional file 1.

Among the identified ORFs, 22 showed high similarity (40 to 82%) with the AtSTP (Sugar Transport Protein) subfamily members. Among these, 5 correspond to the already well known *V. vinifera *hexose transporters named VvHT (*Vitis vinifera *hexose transporter) such as VvHT1, VvHT2, VvHT3 (also named VvHT7), VvHT4 and VvHT5 [35-37], (Additional file 1). Therefore, the 17 newly identified ORFs were named VvHT8 to VvHT24. VvHT8 amino acid sequence shows 99.4% similarity with VvHT1 and the main differences between the two nucleotide sequences reside in some single nucleotide polymorphism and in the length of a microsatellite sequence in the 3'UTR region. Considering that the chromosomal location of *VvHT1 *and *VvHT8 *is not determined, it is difficult to conclude if these sequences represent two independent genes, two alleles of the same gene or possibly one single gene. VvHT9 and VvHT10 share 98.5% similarity between each other and around 73% with VvHT11. Interestingly, the three corresponding genes are located in a tandem repeat region, on chromosome 14. In a similar way, *VvHT14 *to *VvHT24 *form a cluster on chromosome 13 and the 11 corresponding amino acid sequences show very high similarity (more than 90%). VvHT20 and VvHT21, which are located nearby on chromosome 13, contain the two first exons and the last exon of a monosaccharide transporter, respectively. A detailed amino acid sequence analysis revealed that these two partial ORFs are wrongly annotated and might constitute a single monosaccharide transporter, in the third exon of which a stop codon (TAG) replaces a tryptophan residue (TGG). It is therefore tempting to suggest that this point mutation at the origin of the false annotation, might be due to a sequencing error, but we can not exclude that it could be real. Finally VvHT22, VvHT23 and VvHT24 are partial MST, whose sequences do not seem to be fully sequenced, missing either the N-ter or the C-ter region, or both. Therefore, considering that VvHT8 might be identical to VvHT1 and that VvHT20 and VvHT21 are probably a single protein, we can estimate that the grape genome might contain 20 putative hexose transporters. In this VvHT subfamily, the exon-intron organization seems to be conserved as all completely sequenced genes contain 4 exons separated by 3 introns with the exception of *VvHT4 *and *VvHT5*. Phylogenetic analysis (Figure 1) reveals that the VvHT subfamily seems to be divided into two subclades, at the basis of which is located VvHT5 and VvHT12 both present on chromosome 5. VvHT12 is located at the basis of a subclade having a bootstrap value of 100 and containing the 10 closely related transporters from chromosome 13 and 9 (VvHT14 to VvHT24). Furthermore, if we exclude VvHT1 and VvHT8, 3 sister-pairs with a strong bootstrap support (≥ 84%) could be identified, VvHT3/VvHT13 (56.8% similarity); VvHT9/VvHT10 (98.5% similarity) and VvHT2/VvHT4 (52.4% similarity). Finally, phylogenetic analysis of *A. thaliana *and *V. vinifera *sugar transporter proteins allowed us to identify six ortholog pairs between both species (Figure 3) such as VvHT1/AtSTP1 (81.9%), VvHT2/AtSTP5 (65.4%), VvHT3/AtSTP7 (77.2%), VvHT4/AtSTP3 (60.8%), VvHT5/AtSTP13 (82%), VvHT13/AtSTP14 (75.6%). Five of these pairs are supported by bootstrap value of 100%.

### *Vitis vinifera *putative Tonoplast Monosaccharide Transporters (VvTMT; subfamily II)

**Figure 4:**
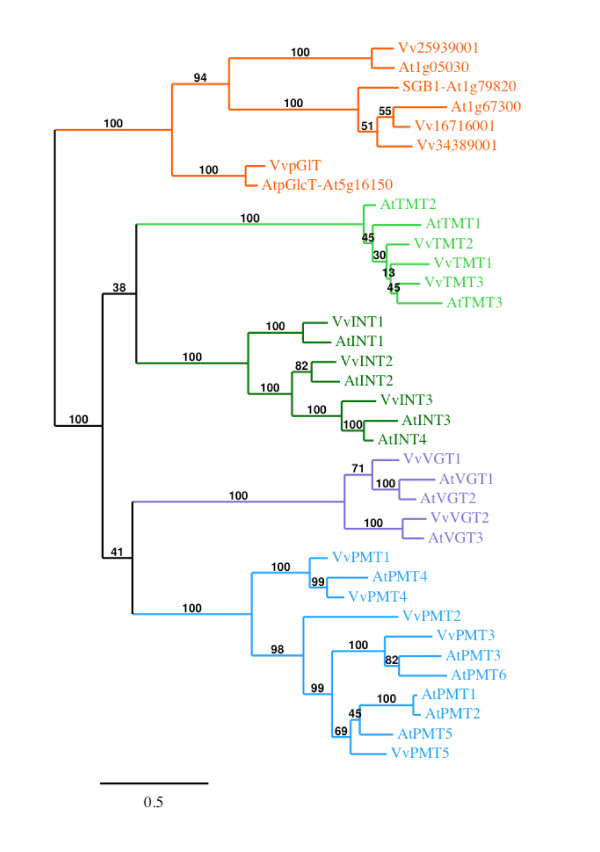
**Maximum likelihood phylogeny of *Vitis vinifera *and *Arabidopsis thaliana *monosaccharide transporter proteins**. The tree was produced using MUSCLE and PhyML with the JTT amino acid substitution model, a discrete gamma model with 4 categories and an estimated shape parameter of 1.448. Bootstrapping was performed with 100 replicates. Accession numbers for *Arabidopsis thaliana *transporters are: At1g20840 (AtTMT1), At4g35300 (AtTMT2), At3g51490 (AtTMT3), At2g43330 (AtINT1), At1g30220 (AtINT2), At2g35740 (AtINT3), At4g16480 (AtINT4), At3g03090 (AtVGT1), At5g17010 (AtVGT2), At5g59250 (AtVGT3), At2g16120 (AtPMT1), At2g16130 (AtPMT2), At2g18480 (AtPMT3), At2g20780 (AtPMT4), At3g18830 (AtPMT5), At4g36670 (AtPMT6); for *Vitis *ones see Additional file 1. *Vitis *ORFs names were simplified, Vv indicating GSVIVT000.

We have also identified three ORFs, which show the strongest similarity (58.3 to 72%) to the 3 *A. thaliana *Tonoplast Monosaccharide Transporters (AtTMT; [44]). All three *Vitis *ORFs show an extended middle loop between the putative trans-membrane helices six and seven in a similar way as the AtTMT. GSVIVT00002919001 is identical to a *V. vinifera *sugar transporter already mentioned in the literature and called VvHT6 [36]. Considering that it shows a higher similarity with AtTMT (58.8 to 70.9%) than with the VvHT proteins (15 to 26.3%), we renamed it VvTMT1. Similarly, GSVIVT00036283001 and GSVIVT00019321001 were called VvTMT2 and VvTMT3, respectively. The exon-intron organization seems to be conserved in the three genes as they contain all 5 exons separated by 4 introns (Additional file 1). Phylogenetic tree performed with *Vitis *sugar transporter amino acid sequences (Figure 1) reveals that the 3 VvTMT form a clade, which is closely related to putative myo-inositol transporters (VvINT) and vacuolar glucose transporters (VvVGT) (described below). Furthermore, phylogenetic analysis using *Vitis *and *Arabidopsis *sequences (Figure 4) confirms that TMT sequences from both species form a single clade, with a strong bootstrap support (100%), but within which low bootstrap values (≤ 45%) indicate unresolved nodes and fail to detect sister-pairs between both species.

### *Vitis vinifera *putative Polyol/Monosaccharide Transporters (VvPMT; subfamily III)

Five ORFs show highest similarity (41.4 to 72.1%) with the 6 *A. thaliana *polyol transporters and have been therefore named VvPMT1 to VvPMT5. *V. vinifera *putative polyol transporter amino acids sequences share 40% to 76.8% similarity between themselves and the corresponding genes present all the same structure with 2 exons separated by a single intron. Phylogenetic analysis performed with the *A*. *thaliana *and *V. vinifera *polyol transporters (Figure 4) reveals that VvPMT1 and VvPMT4 form with AtPMT4 a separated clade. VvPMT2 is at the basis of a second clade, which can be divided into two groups, one including VvPMT3, AtPMT3 and AtPMT6 and the second AtPMT1, AtPMT2, AtPMT5 and VvPMT5. Only AtPMT4 and VvPMT4 could be identified as putative orthologs.

### *Vitis vinifera *putative ERD6-like Transporters (subfamily IV)

**Figure 5:**
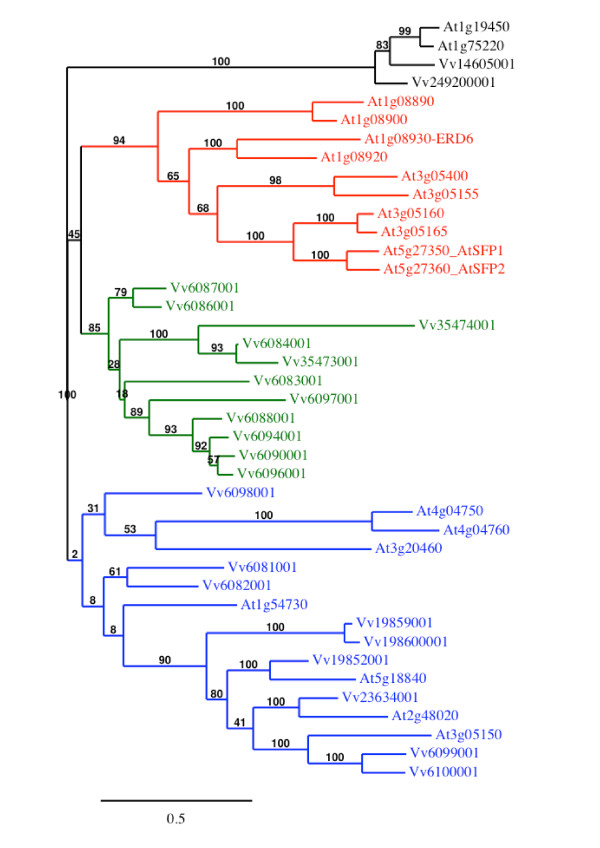
**Maximum likelihood phylogeny of *Vitis vinifera *and *Arabidopsis thaliana *ERD6-like transporter proteins**. The tree was produced using MUSCLE and PhyML with the JTT amino acid substitution model, a discrete gamma model with 4 categories and an estimated shape parameter of 1.404. Bootstrapping was performed with 100 replicates. Arabidopsis transporters are indicated with complete ORFs names, for Vitis ones ORFs names were simplified, Vv indicating GSVIVT000.

Twenty-two ORFs showing strongest similarity with the 19 AtERD6-like proteins were identified (Additional file 1) and share 36.2 to 93.2% similarity with each other. Among them, 6 ORFs correspond to partial sequences in which either the beginning or the end of the protein are not clearly identified. However after a more precise sequence analysis, we were able to realize the full annotation for GSVIVT00006084001 and GSVIVT00006097001. Fourteen ORFs are located on chromosome 14, in a region of tandem gene duplications, three other ORFs are carried by chromosome 5 and two partial ORFs by chromosome 12. The 22 ERD6-like proteins fall into the same subfamily supported by a strong bootstrap value (99%) and 14 loci formed 7 sister pairs (Figure 1). The phylogenetic analysis of the amino acid sequences of ERD6-like transporters from *A. thaliana *and *V. vinifera *(Figure 5) reveals that these transporters can be classified into 4 major groups. A first group includes 7 AtERD6-like located on the 5 chromosomes of *A. thaliana *and 9 VvERD6-like located on 3 chromosomes (5, 7, 14) of *V. vinifera*. A second small group including transporters from both species (At1g19450, At1g75220, Vv14605001 and Vv249200001) was also identified. Inversely, the two last groups include protein sequences from only one species. The *Arabidopsis *group contains 10 proteins located on three different chromosomes (1, 3, 5) including ERD6 [45,46], SFP1 and SFP2 [47]. The *Vitis *group includes 11 proteins, 9 of which are encoded by genes located on chromosome 14.

### *Vitis vinifera *putative Vacuolar Glucose Transporters (VvVGT; subfamily V)

Two *Vitis *ORFs, named VvVGT1 and VvVGT2, show the highest similarity with the 3 AtVGT (Vacuolar Glucose Transporter)-like transporters. In *Arabidopsis *AtVGT1 and AtVGT2 have been shown to be localized in the tonoplast and glucose transport activity has been demonstrated for AtVGT1 [48]. On the contrary AtVGT3 is postulated to be localized in chloroplast membrane as this protein presents a N-terminal extension carrying a potential signal for plastid targeting. Phylogenetic tree (Figure 4) indicates clearly that VvVGT1 is the closest to AtVGT1 and AtVGT2 and that VvVGT2, which presents a N-terminal extension, is more closely related to AtVGT3.

### *Vitis vinifera *putative Inositol Transporters (VvINT; subfamily VI)

We identified 3 ORFs showing the strongest similarity with the 4 AtINT (Inositol transporter) already described in *Arabidopsis*. To our knowledge, only two AtINT have been already characterized. AtINT4 is described as a high-affinity, plasma membrane-localized H^+^/symporter specific for myo-inositol [49]. AtINT1 is a tonoplast-localized H^+^/inositol symporter that mediates the efflux of inositol that is generated during the degradation of inositol-containing compounds in the vacuolar lumen [50]. The three *Vitis *ORFs were named VvINT1-3 according to their highest similarity with AtINT (Figure 4).

### *Other Vitis vinifera *putative monosaccharide Transporters (VvpGlcT/VvSGB1; subfamily VII)

Finally, 4 ORFs show high similarity with the members of the *Arabidopsis *AtpGlcT/AtSGB1 subfamily, which includes proteins showing homology with a putative glucose transporter (pGlcT) of the chloroplast inner envelope membrane from spinach [4] and with a Golgi-localized hexose transporter homolog (suppressor of G protein beta1:SGB1; [6]). The ORF GSVIVT00038247001 is identical to a *V*. *vinifera *sugar transporter already mentioned in the literature and called VvpGlT [20,36]. Phylogenetic tree (Figure 4) reveals that inside this subfamily, the proteins separate into 3 groups having strong bootstrap support (100%). VvpGlT and AtpGlcT fall into the same group, which includes also SopGlcT from spinach (not shown). This observation can argue in favor of a chloroplastic localization of VvpGlT even if the precise localization of this transporter is not demonstrated. In a similar way, the fact that Vv16716001 and Vv34389001 form a second group with At1g67300 and SGB1 indicates that these two *Vitis *putative transporters could be localized in Golgi apparatus. Finally Vv25939001 forms a third group with At1g05030.

### Search for *cis*-elements putatively involved in the transcriptional regulation of sugar transporter genes

We have identified a 2 kb promoter region for each of the 29 fully sequenced genes from the four mostly studied sugar transporter families: VvSUC/SUT, VvHT, VvTMT and VvPMT (Additional file 2). For only three genes *VvHT14 *(1455 bp), *VvTMT3 *(1619 bp) and *VvPMT2 *(623 bp), the identified sequence is shorter due to the presence of an other ORF located less than 2kb upstream of these transporter genes. A PLACE analysis has been applied to these promoter sequences and the 216 identified *cis*-acting elements have been classified per sugar transporter subfamily, for comparison.

#### Cis-elements common to all promoters

In a first approach, 20 common *cis*-regulatory elements conserved in the promoter regions of the 29 analyzed sequences have been identified (Table 1). Only the shortest promoter *VvPMT2 *(623pb) is missing three of these elements, namely CIACADIANLELHC, PYRIMIDINEBOXOSRAMY1A and WBOXATNPR1. Moreover, these common consensus sequences are highly repetitive displaying up to 45 copies into a 2 kb promoter. This might be due to the their limited size (4 to 7 bases), and to their high variability (1, 2 and 3 degenerated nucleotides per motif of 5, 6 and 7 bases, respectively). These common *cis*-acting elements are able to confer expression in distinct plant organs, such as leaves, shoots, roots, seeds, and flowers (pollen). They are also responsive to different plant hormones (abscisic acid, gibberellins, ethylen, cytokinins), as well as to several environmental factors (light, CO_2_, biotic and abiotic stresses). At least a quarter of these common consensus sequences (EBOXBNNAPA, GATABOX, GT1CONSENSUS, GTGANTG10, IBOXCORE) are required for the transcriptional regulation by light, and this mainly in leaves and shoots. This is in agreement with the roles of the studied transporters in sugar allocation between source- and sink-organs. Finally, the presence of the box CIACADIANLELHC, absent only in the *VvPMT2, VvTMT3 *and *VvHT12 *promoters, strongly suggests the importance of circadian regulation for sugar transporter gene expression.

**Table 1:** Common putative *cis*-acting elements identified in the *VvSUC/VvSUT*, *VvHT*, *VvTMT *and *VvPMT *promoter sequences

*Cis*-element name	Sequence	Response	Maximum number of copies/promoter
ARR1AT	NGATT	Cytokinines	36
CIACADIANLELHC	CAANNNNATC	Circadian expression	_4_**
DOFCOREZM	AAAG	C-metabolism, leaf	40
EBOXBNNAPA	CANNTG	Light, ABA, seeds	36
EECCRCAH1	GANTTNC	CO_2_-responsive	7
GATABOX	GATA	Light, leaf, shoot	29
GT1CONSENSUS	GRWAAW	Light, leaf, shoot	39
GTGANTG10	GTGA	Pollen	19
IBOXCORE	GATAA	Light, leaf, shoot	13
MYBST1	GGATA	Myb trans activator	4
MYCCONSENSUSAT	CANNTG	ABA, abiotic stress	36
PYRIMIDINEBOXOSRAMY1A	CCTTTT	Sugar repression, seeds	9*
POLLEN1LELAT52	AGAAA	Pollen	28
RAV1AAT	CAACA	Root, rosette leaves	9
ROOTMOTIFTAPOX1	ATATT	Root	45
SEF4MOTIFGM7S	RTTTTTR	Seed, storage protein	23
WBOXATNPR1	TTGAC	Desease resistance	8*
WBOXHVISO1	TGACT	Sugar, SUSIBA2	9
WBOXNTERF3	TGACY	Wounding, ERF3	16
WRKY71OS	TGAC	GA repressor, ABA	25

Promoter sequence analysis was performed *via *PLACE. *Cis*-element name, sequence motifs and signalling pathway are presented. Up to copies/promoter indicates the highest number of *cis*-acting element found in one promoter. * indicates that the motif is found in all promoters except *VvPMT2*. ** indicates that the motif is found in all promoters except *VvPMT2*, *VvTMT3 *and *VvPMT2*.

#### Cis-elements present only in a single promoter

A second complementary approach was targeted to unique consensus sequences present in the promoter of only one sugar transporter gene, thereby implying some expression specificity. The few unique identified *cis*-elements (Table 2) are characterized by longer sequences (5 to 10 bases), and usually lacking any nucleotide variability. Interestingly, among the 9 gene specific motifs identified, 4 are present only in the *VvHT5 *promoter, 2 in *VvTMT3*, one in *VvHT2 *and another one in *VvSUC11*. This means that a limited number of gene specific *cis*-acting elements is concentrated in the promoter regions of few sugar transporter genes. For example the hexose transporter gene *VvHT5 *is the only one among the 29 genes studied, displaying 4 unique motifs (ABREZMRAB28, CRTDREHVCBF2, GBOXLERNCS, LREBOXIIPCCHS1) in its promoter. Finally, another specific *cis*-element strongly restricted to *VvSUC/VvSUT *genes is the motif MYBCOREATCYCB1 (Table 2), required for transcriptional regulation of cyclin B1 at two different phases of the cell cycle, G1/S and G2/M transitions [51].

**Table 2:** Unique *cis*-acting elements identified only in the promoter sequence of a single sugar transporter gene

*Cis*-element name	Sequence	Response	Copy number	Gene
ABREZMRAB28	CCACGTGG	Drought, ABA	2	HT5
CRTDREHVCBF2	GTCGAC	Cold, drought	2	HT5
GARE2OSREP1	TAACGTA	GA, germination	1	TMT3
GBOX10NT	GCCACGTGCC	Leaf, root, flower, pollen	1	HT2
GBOXLERNCS	MCACGTGGC	Light, overlap ABA	1	HT5
LREBOXIIPCCHS1	TCCACGTGGC	Cold, drought, ABA	1	HT5
MYBCOREATCYCB1	AACGG	cell cycle, cyclin	1 to 3	VvSUC/VvSUT
NONAMERMOTIFATH3H4	CATCCAACG	meristem,	1	TMT3
ZDNAFORMINGATCAB1	ATACGTGT	Light, leaf, shoot	1	SUC11

Promoter sequence analysis was performed *via *PLACE. *Cis*-element names, sequence motifs, signalling pathways and the number of copies for each element are presented. Gene indicates the corresponding gene in which the *cis*-elements are found.

#### Cis-elements involved in sugar regulated transcription

We have studied the transcriptional regulation of sugar transporter genes through the repertory of the main promoter motifs potentially involved in sugar-regulated transcription, and this in combination with other metabolic and hormonal signalling. Additional file 3 summarizes the careful comparison of the following consensus sequences: *i) *elements for sugar responsiveness as the SURE boxes [52], the bipartite sucrose box 3 [53], the CGACGOSAMY3 [54], the CMSRE [55], the SP8 and WBOXHVISO1 sequences enabling the binding of some WRKY-type proteins at the example of SPF and SUSIBA2 [56-58]; *ii) *sequences common for hormonal and metabolic (sugar) signals perception as the S-box for sugar and ABA [59], the MYBGAHV for gibberellins (GAs) induction and sugar repression [60]; the GARC complex consisting of the AMYBOX1 and 2 [61], and PYRIMIDINE boxes for GAs, ABA and sugar regulation.

There is at least one gene for each subfamily displaying the majority of chosen sugar responsive motifs (*VvHT1 *and *VvHT8 *- 9 motifs, *VvSUC11 *and *VvSUT2 *- 8 motifs, *VvHT5, VvTMT3 *and *VvPMT5 *- 7 motifs), thus suggesting a possible transcriptional control dependent on sugars as metabolic signals (Additional file 3). The sucrose box 3, is the most frequently found *cis*-acting element, present at 1 to 4 copies in all studied promoters except *VvHT15*, *VvTMT1 *and *VvPMT2*. On the contrary, the CMSRE1IBSPOA element, involved in sucrose positive regulation is only found in promoter regions of *VvHT2*, *VvHT5 *and *VvTMT3*. The sucrose transporter gene family, is the only one displaying the SURE2 motif in the promoter regions of *VvSUC11 *and *VvSUC27*. The sugar responsiveness CGACGOSAMY3 box is carried only by VvHT genes (*VvHT1*, *VvHT3*, *VvHT5*, *VvHT8*, *VvHT11*), and not by the other subfamilies. Similarly, the S-box (CACCTCCA) usually closely associated to the light-responsive G-box, is carried also only by *VvHT *genes, namely *VvHT1*, *VvHT8 *and *VvHT11*. Inversely, the motif MYBGAHV involved in sugar and GA signalling pathways, is displayed by *VvSUC/SUT*, *VvPMT *and *VvTMT *genes, but is lacking in *VvHT *ones. Finally it appears that *VvHT1 *and *VvHT8 *promoter sequences are the only one to contain a putative GARC complex. A more detailed comparison into the *VvHT *subfamily reveals that *VvHT1 *and *VvHT8 *promoters are carrying the same *cis*-elements (with the exception of one more copy of the PYRIMIDINEBOXHVEPB1 for *VvHT1*). This indicates that as the coding sequences, the promoter regions for these two putative genes present also a very high similarity (96.7%). This argues in favor of the assumption that these are either alleles of the same gene or represent the same gene, as already suggested in this study. Such consideration is valuable for another couple of genes mentioned above, *VvHT9 *and *VvHT10*, sharing a strong sequence similarity, displaying the same *cis*-acting elements in their promoter region, and carried on the same chromosome. A third gene *VvHT11 *is present in tandem repeat with both *VvHT9 *and *VvHT10*, by the same chromosome, thus suggesting that they may be products of successive duplications.

### *V. vinifera *sugar transporter genes expression in vegetative organs

**Figure 6:**
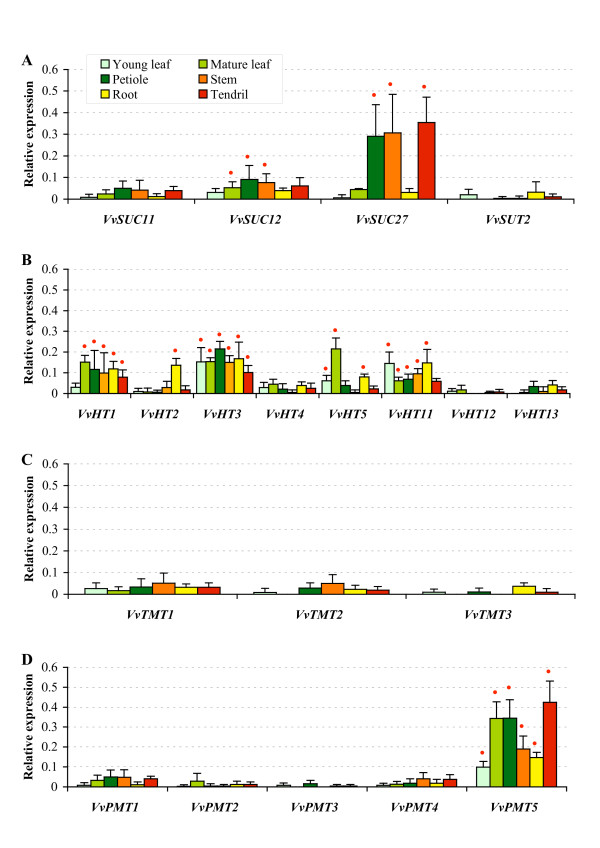
**Macroarray analysis of *VvSUC*/VvSUT (A), *VvHT *(B), *VvTMT *(C) and *VvPMT *(D) genes expression in grapevine vegetative organs**. RNA was isolated from vegetative organs collected from 20 independent plants. Gene transcript levels were normalized against four reference genes (*GAPDH*, *EF1α*, *EF1γ*, *actin*). Each value represents the mean of six replicates obtained with two independent experiments. Red point indicates an expression value higher than the mean of the expression value for all genes in the tested organ (mean value of relative expression for young leaf: 0.03; mature leaf: 0.05; petiole: 0.07; stem: 0.05; root: 0.05; tendril: 0.07).

In order to study the expression pattern of grapevine sugar transporter genes identified above and belonging to the *VvSUC/VvSUT, VvHT*, *VvTMT*, and *VvPMT *subfamilies, we have developed sugar transporter macroarray membranes. Specific regions for each sugar transporter (Additional file 4) have been identified in the 3'UTR of the corresponding nucleotide sequences, amplified by PCR using Chardonnay genomic DNA and spotted on nylon membrane. *VvHT8, VvHT9, VvHT10 *and *VvHT14 *to *VvHT24*, could not be considered in this expression analysis, as it was not possible to found specific DNA region for these transporters, due to their high sequence similarity either with *VvHT1 *or between each other. To determine the gene expression patterns in vegetative organs, macroarrays were hybridized with ^33^P-labelled first-strand cDNA synthesized from total RNA isolated from young leaves, mature leaves, petioles, stems, roots and tendrils from 10 weeks-old grapevine plants grown under aeroponic conditions. These culture conditions have been used in order to collect all main vegetative organs at the same development stage and in the same conditions and allowed an easy access to the root system without damage. Among the sucrose transporters only *VvSUC27 *is detected at a high level in petioles, stems and tendrils, its transcripts being less abundant in young leaves, mature leaves and roots (Figure 6A). *VvSUC11 *and *VvSUC12 *are detected in all organs but at a weaker level and *VvSUT2 *is the less expressed sucrose transporter being only weakly detected in young leaves and roots. Concerning the *VvHT *family (Figure 6B), 3 genes (*VvHT1*, *VvHT3 *and *VvHT11) *are expressed in all the tested organs at a relatively high level (expression value higher than the mean of expression value for all genes) with *VvHT1 *and *VvHT11 *being less expressed in young leaves and tendrils, respectively. *VvHT2 *and *VvHT5 *seem to present a more specific expression. *VvHT2 *is expressed at a higher level in roots than in the other organs and *VvHT5 *is highly expressed in mature leaves and presents a weaker expression in roots and young leaves. The three other hexose transporters (*VvHT4*, *VvHT12*, *VvHT13*) are weakly detected, indicating a low expression in the tested organs. The three *VvTMT *are also detected at a very low level in all organs (Figure 6C). Among the polyol/monosaccharide transporters (Figure 6D), only *VvPMT5 *could be significantly detected in the six organs. It shows a strong expression in mature leaves, petioles and tendrils, and a weaker expression in stems, roots and young leaves. The other *VvPMT *are weakly expressed in all the tested organs.

**Figure 7:**
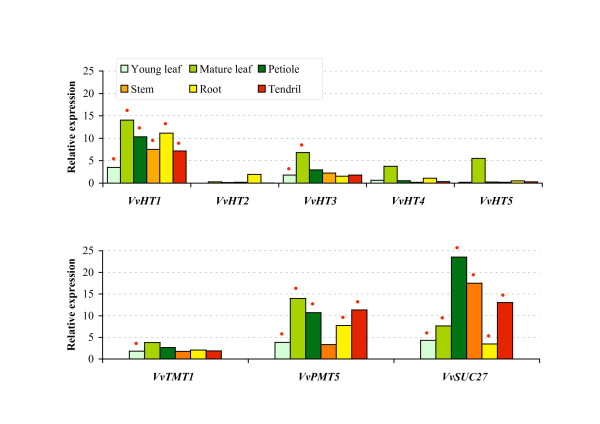
**RNA gel blot analysis of *V. vinifera *sugar transporter genes transcript levels in vegetative organs**. RNA was isolated from vegetative organs collected from 20 independent plants. Gene transcript levels were quantified using Image Quant 5.2 software and normalized against *GAPDH *gene expression. Red point indicates an expression value higher than the mean of the expression value for all genes in the tested organ (mean value of relative expression for young leaf: 1.65; mature leaf: 5.62; petiole: 6.01; stem: 3.55; root: 3.37; tendril: 4.10).

In order to validate the results obtained with macroarray hybridizations and to confirm the expression pattern of the sugar transporter genes, we performed a Northern blot analysis for few genes. The results presented in Figure 7 clearly show similar expression patterns for most of the tested genes. *VvHT1 *shows the weakest expression in young leaves. *VvHT2 *seems to be specifically expressed in roots. *VvHT5 *presents a weak expression in all organs, except in mature leaves. *VvTMT1 *shows a global weak expression in all organs. *VvPMT5 *is highly expressed in mature leaves, petioles and tendrils. Finally, *VvSUC27 *shows high amount of transcripts in petioles, stems and tendrils. Few discrepancies were however observed. First, the expression level detected for *VvHT3 *is the highest in mature leaves when detected by Northern blot which is not the case using macroarray. Second, *VvHT3 *and *VvHT11 *show a higher expression in macroarray than in Northern blot analysis. For *VvHT11 *signals obtained with Northern blot were too low to be correctly quantified. Furthermore, we could confirm using both methods that *VvHT3 *is expressed at a higher level than *VvHT2*, *VvHT4*, *VvHT5*, and *VvHT11 *in almost all organs. Taken together, all these results indicate that few transporter genes (*VvHT1*, *VvHT3*, *VvPMT5*, *VvSUC27*) are the most expressed in almost all vegetative organs and that *VvHT2 *and *VvHT5 *are more specifically expressed in roots and mature leaves, respectively.

### Sugar transporter genes expression during grape berry development

**Figure 8:**
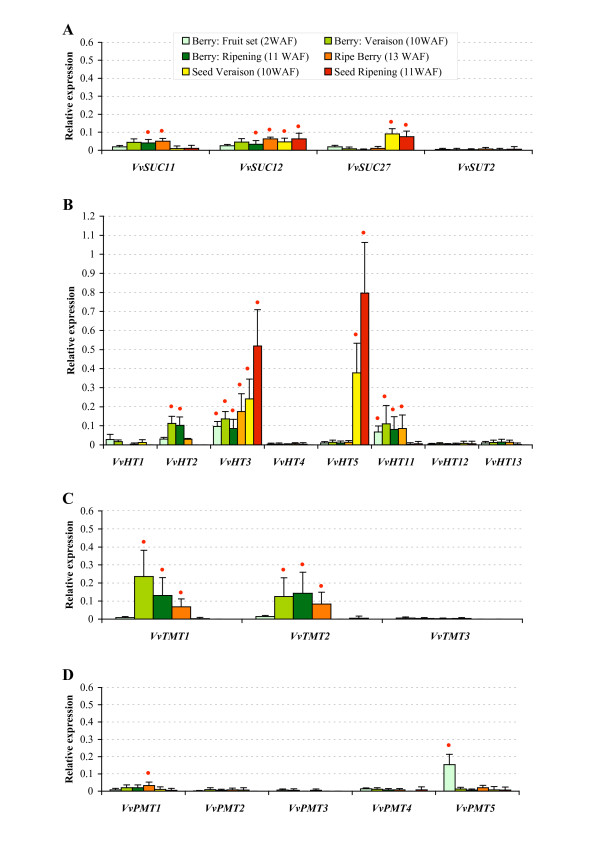
**Macroarray analysis of *VvSUC/VvSUT *(A), *VvHT *(B), *VvPMT *(C), *VvTMT *(D) genes expression during grapevine berry and seed development**. For each developmental stages, RNA was isolated from all berries collected from 5 independent grapes. Gene transcript levels were normalized against four reference genes (*GAPDH*, *EF1α*, *EF1γ*, *actin*). Each value represents the mean of six replicates obtained with two independent experiments.. Red point indicates an expression value higher than the mean of the expression value for all genes in the tested organ (mean value of relative expression for berry 2 WAF: 0.056; berry 10 WAF: 0.046; berry 11 WAF: 0.033; berry 13 WAF: 0.029; seed 10 WAF: 0.032; seed 11 WAF: 0.058).

In order to study the expression of sugar transporter genes during berry development, we further hybridized the sugar transporters macroarray membranes with ^33^P-labelled first-strand cDNA synthesized from total RNA isolated from berries and seeds. Four developmental stages for berries - fruit set (2WAF), veraison (10WAF), ripening (11WAF), ripe berries (13WAF) - and two for seeds (10 and 11 WAF) were used. Among the sucrose transporter genes, *VvSUC11 *and *VvSUC12 *are both expressed during berry development at a similar level to that detected in vegetative organs (Figure 8A). The weakest expression for these two genes is observed in berries at the stage of fruit set. On the contrary, *VvSUC27*, the most expressed sucrose transporter gene in vegetative organs is less expressed than *VvSUC11 *and *VvSUC12 *in berries while *VvSUT2 *is weakly or not detected. Three hexose transporters are expressed during berries development (Figure 8B). *VvHT2 *is expressed during the 4 tested stages and presents a maximum at the veraison and during ripening. Inversely, *VvHT3 *and *VvHT11 *are expressed at an equivalent level during the 4 developmental stages. *VvHT1*, *VvHT4*, *VvHT5*, *VvHT12 *and *VvHT13 *are poorly or not detected at any stage. The expression of two putative tonoplast monosaccharide transporters (*VvTMT1 *and *VvTMT2*) which is weak at the fruit set increases significantly at the veraison (figure 8C). Furthermore, the expression level of these two genes is higher in berries at the veraison and during ripening than in vegetative organs. On the contrary, *VvTMT3 *does not seem to be expressed in berries at any stage of development. Polyols transporters are not highly expressed in berries, only *VvPMT1 *is weakly detected during the 4 stages and *VvPMT5 *is expressed mainly at the fruit set stage (Figure 8D). Macroarray hybridization performed with first-strand cDNA synthesized from total RNA isolated from seeds reveals clearly that the expression of *VvHT3 *and *VvHT5 *is very high in seeds and increases during seed development (Figure 8B). Two other transporter genes *VvSUC12 *and *VvSUC27 *were also expressed in seeds but at a lower level, and their expression stays stable during the two tested developmental stages (Figure 8A).

## Discussion

### Phylogenetic analysis of *Vitis vinifera *sugar transporter genes

The search for sugar transporters in the *Vitis vinifera *translated genome has identified 4 sucrose and 59 putative monosaccharide transporters including 20 VvHT (Hexose Transporters), 3 VvTMT (Tonoplastic Monosaccharide Transporters), 5 VvPMT (Polyol/Monosaccharide Transporters), 3 VvINT (INositol Transporter), 2 VvVGT (Vacuolar Glucose Transporters), 4 pGlT/SGB1 and 22 ERD6-like transporters. As expected, phylogenetic analysis performed with these sugar transporter proteins revealed that sucrose and monosaccharide transporters form two distinct groups (Figure 1). This analysis allowed us to identify only 4 *Vitis *sucrose transporters, which confirms that, as all other analyzed plants, *Vitis *possesses a small sucrose transporter gene family, in which one gene (*VvSUC12*) belongs to the SUT2 subfamily. Interestingly, in *Vitis *as in *Arabidopsis*, the VvHT and the ERD6-like form the largest multigenic subfamilies. In *Vitis*, this may be due to the presence of 4 repeated regions, encompassing *VvHT *and *ERD6-like *genes. Two duplicated regions located on chromosomes 13 and 14 contain 9 and 3 *VvHT*, respectively. The 2 other regions carried by chromosomes 5 and 14 display respectively 3 and 14 *ERD6-like *genes. Similarly, in *Arabidopsis*, the large expansion of AtSTP subfamily has been correlated with 3 segmental duplications and one tandem duplication as well as the expansion of the AtERD6-like subfamily by 2 segmental duplications and 6 tandem duplications [16]. Furthermore, based on significant differences in size observed in the STP and ERD6-like subfamilies, between the non-vascular (moss) and the vascular (gymnosperm and angiosperm) lineages, it has been suggested that the expansion of these two subfamilies could be related to the evolution of vascular plants. This is reflecting the increased importance of the sugar transport and sugar transporters in vascular plants [16]. In agreement with this hypothesis, the AtERD6/VvERD6-like phylogenetic tree (Figure 5) clearly shows that ERD6 transporters from both species fall into four different groups, two of them containing either AtERD6 or VvERD6 transporters only. This indicates that in both species, the expansion of the ERD6-like subfamilies has occurred quite recently, after the separation of these two species.

### Sugar-responsive elements in sugar transporter gene promoters suggest their regulation by sugars

The *in silico *search for *cis*-acting elements reveals several common and highly repetitive motifs in sugar transporter gene promoters. These *cis*-acting elements such as DOF (DNA-binging with one finger) proteins, may play a role not only in the regulation of sugar transporter gene expression in terms of activity level, but also plausibly in terms of response specificity *via *a combinatory control. Such a control has already been suggested for *AtSUC2 *[62] the expression of which in the companion cell is regulated by the close cooperation of binding sites for a DOF and a putative HD-Zip transcription factors. Several transporter gene promoters display an important concentration of sugar-responsive elements suggesting their possible transcriptional regulation by sugars. To our knowledge, the transcriptional regulation of *VvHT1 *by glucose is the only one to be clearly demonstrated [18-20] and this is confirmed by the fact that *VvHT1 *promoter contains the highest number of sugar responsive motifs. This highlights the power of the *in silico *analysis as a first step toward the functional characterization of promoter regions. Finally, the MYBCOREATCYCB1 sequence exclusively found in *SUC/SUT *promoters is not surprising in regard to the sucrose-dependent induction of *Cyclin D3 *gene expression [63], thus suggesting a possible concomitant regulation of some sucrose transporter genes in the cell cycle.

### Sucrose transporter genes expression in *Vitis vinifera*

The expression patterns detected for the sucrose transporter genes, using macroarrays, are in good agreement with those described in the literature with two main exceptions: the absence of high expression of *VvSUC11 *in seeds and of *VvSUC27 *in roots, as reported by [30]. On the contrary, we confirmed that, in berries, *VvSUC11 *and *VvSUC12 *transcripts are present at all developmental stages and accumulate slightly at the onset of ripening [30,31]. VvSUC11 is closely related to AtSUC4, which has been localized in the tonoplast of *A. thaliana *mesophyll cells [64]. Furthermore, VvSUC12 falls into the SUT2/SUC3 group which contains very low affinity sucrose transporters for which different putative physiological functions have been proposed including their putative involvement in sucrose import into several sink tissues [43,65,66]. Therefore, it is tempting to suggest that VvSUC12 is probably involved either in phloem unloading or in sucrose import into berry tissues and that VvSUC11 might be responsible for sucrose accumulation in berry vacuoles. This hypothesis will have to be verified with the precise localization of these two transporters. We noticed that *VvSUC27 *is the most expressed sucrose transporter gene in vegetative organs and that its expression is relatively low in berries. Considering its high expression in petioles, stems and tendrils and the fact that it is closely related to members of the SUT1 subfamily, VvSUC27 is probably responsible for phloem loading and sugar retrieval during long-distance transport. Finally, the weak expression level observed for the less characterized *Vitis *transporter gene *VvSUT2 *makes it difficult to assign a specific role for this transporter.

### Hexose transporter genes expression in *Vitis vinifera*

The present phylogenetic analysis indicates that VvHT1 shows highest similarity with AtSTP1. Both are high affinity glucose transporters showing *K_m _*value of 70 μM [36] and 20 μM [67], respectively. During the last decade, different authors have reported various expression patterns for *VvHT1 *such as a strong expression in berries and young leaves [35], a preferential expression in sink organs [18], an expression in conducting bundle of leaves, petioles and berries [36] or an expression increasing with leaf development [37]. During berry development, *VvHT1 *expression was described to show two peaks (one at the time of anthesis, the other after veraison) [35] or to decline rapidly during ripening [37,68]. This second expression pattern was supported by the detection of VvHT1 protein only in young green berries [20]. Our results (Figures 6 and 8) clearly confirm that *VvHT1 *belongs to the hexose transporters that are poorly expressed in berries, but is one of the mostly expressed *VvHT *in vegetative organs including leaves, petioles, stems, roots and tendrils. Furthermore, its expression increases during leaf development.

VvHT2 shows the highest similarity with AtSTP5 which has not been yet characterized. Different reports describing the expression of *VvHT2 *have shown that *VvHT2 *is weakly expressed in leaves whatever the stage of development [35] and that the transcript level is high in young berries and declines around veraison [35,37,68]. Our data (Figures 6 and 8) confirm not only *VvHT2 *expression in leaves and during berry development, but indicate also its weak expression in almost all vegetative organs except for roots in which it seems to be strongly expressed.

*VvHT3 *has been described to be one of the mostly expressed *VvHT *in leaves, with increasing expression during leaf development. In young berries, its expression is high, decreases around veraison and increases again around the phase of sugar storage [37]. The expression pattern determined in our experiment (Figures 6 and 7) correlates with that described previously. However, we found that this transporter, which is expressed in all vegetative organs is also highly expressed in seeds (Figure 8), in which its expression seems to increase during development. Interestingly, *VvHT3 *shows the highest similarity with *AtSTP7 *for which a strong seed expression is also suggested by microarray hybridization data (genevestigator, BAR). Although the localisation and the functionality of these transporters are unknown, they might have a determinant function in sugar storage in seeds and/or in embryo development.

*VvHT4 *is poorly expressed in all the tested organs and hardly detectable in berries (Figures 6 and 8), in accordance with previous report describing a very weak expression in berry and leaf development [37]. VvHT4 has been characterized as a glucose transporter showing a high affinity for glucose (*K_m _*: 137 μM), higher than that reported for AtSTP3 (*K_m _*= 2 mM) the closest related *Arabidopsis *transporter. A physiological role, either to support wounded tissue or in the retrieval of monosaccharides released during cell damage and cell wall degradation, has been proposed for AtSTP3, based on its induction after wounding [2]. A more precise characterization of VvHT4 is therefore required to verify if the expression of this gene is also regulated after wounding or in response to other stresses.

*VvHT5 *was found to be less expressed than *VvHT1 *and *VvHT3 *in developing leaves and during berry development [37]. Our experiments confirmed that *VvHT5 *transcripts are hardly detected in berries, however they are predominant in seeds, at least for the two tested developmental stages (Figure 8). VvHT5 shows the highest similarity with AtSTP13 and both have similar high affinity for glucose (*K_m _*= 89 μM and *K_m _*= 74 μM, respectively) [37,69]. Furthermore, the expression of these two transporters is described to be induced in response to pathogen attack [69,70]. This indicates that these genes could be involved in pathogen starvation and/or in a sugar signalling pathway in plant defense. The presence of four unique *cis*-acting elements (Table 2) and of a cluster of three ABRE motifs [70] in the promoter region of *VvHT5 *gene is in agreement with the regulation of its expression by ABA and biotic stress but also suggests its involvement in abiotic stress responses.

Three putative hexose transporters named *VvHT11*, *VvHT12 *and *VvHT13*, that have never been described earlier, have been identified. Our phylogenetic analysis (Figure 3) revealed that VvHT11 and VvHT12 are each located at the basis of one of the two duplicated regions involved in the expansion of the VvHT subfamily. Furthermore, this analysis allowed us to identify AtSTP14 and VvHT13 as orthologs, but no orthologs for VvHT11 and VvHT12 could be found. The expression pattern of *VvHT11*, the weak expression of *VvHT12 *and *VvHT13 *in all vegetative organs and in berries are not sufficient to suggest putative physiological functions for these three transporters. However recent data indicates that *VvHT13 *is induced by necrotrophic fungi and could be involved as *VvHT5 *in biotic stress response (Afoufa-Bastien et al., personal communication).

### Putative tonoplastic monosaccharide transporter genes expression in *Vitis vinifera*

Three putative tonoplastic monosaccharide transporters were identified and named VvTMT1-3. Expression data indicate that, even if the expression of *VvTMT1 *and *VvTMT2 *is present in all the tested vegetative organs, it is highest in developing berries. The expression of VvTMT1 is described to increase during berry development [71] with a maximum near the start of veraison [68]. Our data not only confirm this expression pattern but suggest also that at least two transporters, VvTMT1 and VvTMT2, might probably play a significant role during ripening. Although their cellular localisation and their transport activity have not been determined so far, they could be involved in hexose accumulation into vacuoles of berry cells. Furthermore, considering that the expression of *AtTMT1 *and *AtTMT2 *has been reported to be induced by drought, salt, and cold treatments [44], it would be interesting to verify, if the expression of the *VvTMT *genes is also regulated under stress conditions, particularly in vegetative organs, where they are weakly expressed in normal conditions.

### Polyols transporter genes expression in *Vitis vinifera*

Since 2001, many polyol transporters have been identified and characterized in sorbitol or mannitol-translocating plants, where they are described to be responsible for the loading of polyols into the phloem and their transfer to sink organs [11,12,38-40]. More recently, polyol transporters have been identified and characterized in non-polyol-translocating species such as *A. thaliana *[72-74] which contains 6 polyol transporters, the physiological role of which is still unknown. In *Vitis*, the expression of only one EST encoding a putative PMT has been already briefly mentioned in the literature and was shown to be weakly expressed during berry development [68]. Our *in silico *analysis, indicates that the *Vitis *genome contains 5 putative polyol transporter genes. Among them only one named *VvPMT5 *was highly expressed in vegetative organs and only at the fruit set. However as grapevine has not been described as a species transporting polyols in the phloem, the role of this transporter is far from being clear.

## Conclusions

**Figure 9:**
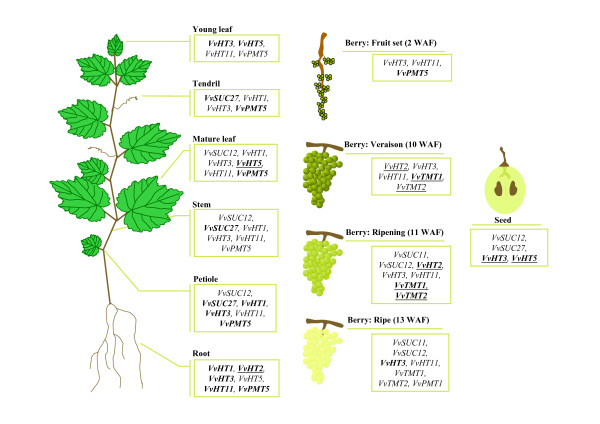
**Schematic representation of preferential expression of *Vitis vinifera *sugar transporter genes in the different vegetative organs and during berry development**. This summary is based on the expression data described in the present report. For each organ, only genes with an expression value higher than the mean of the expression value for all genes are presented. Genes indicated in bold are the most expressed in the indicated organ. Underlined genes are those showing a preferential expression or being induced during the development of the considered organ.

The present work represents the first exhaustive analysis of sugar transporter genes in a woody plant. The identification of grapevine sugar transporter genes and their comparative analysis with the *Arabidopsis *ones has indicated a strong conservation of these genes between herbaceous and woody plants as well as some expansions of particular functional subfamilies such as hexoses and ERD6-like transporters. In this paper, we developed macroarrays to profile the expression of 20 of these transporters simultaneously in different organs. Our results not only confirmed some expression data already described in the literature but also demonstrated that four sugar transporter genes are expressed in almost all vegetative organs (*VvHT3*, *VvHT11*, *VvPMT5, VvSUC27*), few transporters are more specifically expressed in roots (*VvHT2*), mature leaves (*VvHT5*) and/or in seeds (*VvHT3*, *VvHT5*) and three others are regulated during berry development (*VvHT2*, *VvTMT1*, *VvTMT2*) (Figure 9). The present results might help to elucidate the biological function of sugar transporters in *V. vinifera *development particularly during berry ripening and would also have a significant impact on our knowledge on plant sugar transporters in general. The *in silico *analysis of promoter sequences revealed the presence of *cis*-regulatory elements involved in sugar signalling, and represents a first step towards the understanding of the regulation of sugar transporter gene expression *via *metabolic, hormonal and environmental signals. More and more evidences suggest that sugar transporter genes are regulated under various conditions. Thus, the macroarray analysis described in this paper could constitute a powerful approach to investigate the sugar transporter response to environmental factors in grapevine.

## Methods

### Identification of sugar transporter genes in *V. vinifera *genome

*V. vinifera *sugar transporter genes were identified performing a Blastp analysis [75] against the *V. vinifera *proteome 8X database, on Genoscope website http://www.genoscope.cns.fr/externe/GenomeBrowser/Vitis using each *A. thaliana *monosaccharide and sucrose transporter amino acid sequences as query, and an E-value of 1,00^E-04 ^as threshold. Furthermore, the 2 kb region upstream of the start codon for each gene was considered as the promoter sequence.

### Sequence similarities, phylogeny and promoter sequence analysis

Sequence similarities were determined performing Clustal V multiple alignments using Lasergene software (DNASTAR, USA). Phylogenetic analysis of *V. vinifera *and *A. thaliana *sugar transporter protein sequences was performed using maximum likelihood and the http://www.phylogeny.fr website[76]. For this, protein sequences alignment was performed using the MUSCLE program [77], and maximum likelihood trees with 100 bootstrap replicates were constructed with the PHYML program [78,79] and the JTT amino acid substitution model. Phylogenic tree was visualized using Treedyn program [80]. Search for *cis*-regulatory elements in promoter sequences was performed using the PLAnt Cis acting regulatory DNA Elements database (PLACE: http://www.dna.affrc.go.jp/PLACE/index.html)

### Plant material and growth conditions

*Vitis vinifera *cv. Chardonnay plants were cultivated *in vitro*, for 7 weeks (46 days), on McCown Woody Plant Medium (Duchefa, The Netherlands), pH 5.8, supplemented with 20 g.l^-1 ^sucrose, with 16 h photoperiod at 24°C. Plants were then transferred to an aeroponic culture system and grown with Gibeaut solution [81] under controlled conditions (16 h photoperiod, 23°C; 70% RH day/18°C; 65% RH night). After 24 days, young and mature leaves, stems, roots, petioles and tendrils were sampled, immediately frozen in liquid nitrogen and stored at -80°C. *V. vinifera *cv. Chardonnay berries were harvested in the 2007 growing season (between 25^th ^June and 10^th ^September) from grapevines grown in SRPV Poitou-Charentes fields (Biard, Poitiers, France). Berries were sampled at 2, 10, 11 and 13 weeks after flowering (WAF) corresponding to fruit set, veraison, and two maturation developmental stages, the last one being 10 days before harvest. After freezing in liquid nitrogen, seedless berries and seeds were stored at -80°C.

### RNA isolation

Total RNA was isolated from grapevine tissues as previously described by Valtaud and coworkers (2009) [82]. For macroarray analysis, RNA was treated with RNase-free DNaseI (QIAGEN, Germany) in order to eliminate contaminant DNA and purified using the RNeasy Mini Kit (QIAGEN, Germany), according to the RNA clean up protocol.

### Cloning of specific cDNA fragments and macroarray membrane spotting

Specific DNA regions for each sugar transporter and reference genes (*actin, EF1α*, *EF1γ *and *GAPDH*) have been identified in the 3'UTR of the nucleotide sequence and amplified by PCR using Chardonnay genomic DNA and specific primers (Additional file 4). PCR products were purified with Wizard^®^SV Gel and Clean-Up System (Promega, USA) according to manufacturer's protocol, cloned into the pGEM^®^-T Easy Vector (Promega, USA) and sequenced using the ABI PRISM^® ^BigDye^® ^Terminator v3.1 Cycle Sequencing Kit (Applied Biosystems, USA).

Specific cDNA fragments have been amplified from the obtained plasmids by PCR using one specific primer and T7 primer. For each reaction, 1 μl of plasmid DNA solution was used as template in a 50 μl PCR reaction, containing 1× Green GoTaq^® ^Flexi Buffer, 2 mM MgCl_2_, 0.4 μM of each primer, 0.2 mM of each deoxynucleotide and 1.25 U of GoTaq^® ^DNA Polymerase (Promega, USA). Amplification reactions included an initial denaturation step at 94°C for 5 min, followed by 30 cycles of 1 min at 94°C, 1 min at 52°C, 1 min at 72°C and a final extension of 5 min at 72°C. All PCR products were purified using the Wizard^®^SV Gel and Clean-Up System (Promega, USA) according to manufacturer's protocol.

Each cDNA fragment was dotted in triplicate on a 6× SSC-soaked nylon Hybond™-N^+ ^membrane (GE Healthcare, UK), using a 96-well Bio-Dot^® ^Microfiltration Apparatus (BIO-RAD, Canada). The amount of cDNA per spot was 50 ng for sugar transporter and 100 ng of each reference gene. Three dots of 50 ng of salmon sperm DNA were used as internal negative control. Membranes were then incubated in a denaturing solution (1.5 M NaCl, 0.5 M NaOH), in a neutralizing solution (1.5 M NaCl, 0.5 M TRIS-HCl pH 8), and finally washed in 2× SSC solution. DNA was cross-linked to the membrane by exposure to UV light (120 mJ/cm^2^) using a crosslinker (Bio-Link-BLX-E254).

### Macroarray membrane hybridization

For the synthesis of ^33^P-labeled cDNA, 30 μg of DNase treated total RNA were retro-transcribed using 2 μM oligo d(T)_16_, 0.5 mM dATP, dTTP, dGTP, 2.26 μM dCTP, 0.33 μM [α-^33^P]-dCTP (10mCi ml^-1^) and 800 U of M-MLV Reverse Transcriptase (Promega, USA). Labeled products were then treated with 10 U of Ribonuclease H (Promega, USA) and purified on illustra™ Probe Quant™ Micro Columns (GE Healthcare, UK). Prehybridization and hybridization were carried out at 65°C using Church solution (1% BSA, 1 mM EDTA, 0.25 M Na_2_HPO_4_-NaH_2_PO_4 _and 7% SDS). After 16-20 h of hybridization, membranes were washed twice in 2× SSC; 0.1% SDS for 15 min, twice in 1× SSC; 0.1% SDS for 15 min at 65°C and exposed in a Storage Phosphor Cassette for 48 h and images were acquired using a Typhoon TRIO Imager (GE Healthcare, UK). Spot finding, quantification and background subtraction were done with ImageQuant TL 7.0 program (GE Healthcare, UK). Spots were considered as present only if higher than the mean of salmon sperm negative control and then normalized using the mean of 4 reference genes (*actin, EF1α, EF1γ *and *GAPDH*).

### Northern blotting

Total RNA (20 μg) isolated from different organs of grapevine plants were separated by electrophoresis in denaturing formaldehyde 1.2% agarose gel and then transferred to Hybond™-N Nylon membrane (GE Healthcare, UK). DNA probes designed on 3'UTR regions of genes of interest were produced by PCR reaction and labeled with [α-^32^P]-dCTP using Prime-a-Gene^® ^Labelling System (Promega, USA) according to manufacturer's protocol. Prehybridization and hybridization were performed as described for macroarray. Membranes were washed in 2× SSC, 0.1% SDS for 15 min, in 1× SSC, 0.1% SDS for 15 min and in 0.5× SSC, 0.1% SDS for 5 min at 65°C. Membranes were exposed for 48 h in a Storage Phosphor Cassette and scanned as performed for macroarray analysis. Quantification and background correction were done using Image Quant 5.2 software. Reported signals were then normalized to *GAPDH *expression value.

## Authors' contributions

DAB and AM were equally involved in plant culture, RNA isolation, cDNA cloning, macroarrays and Northern blot hybridizations, data analysis and participated in genome search and manuscript writing. JJ participated in genome search, cDNA cloning and macroarrays perfecting. PCT helped in macroarrays perfecting and was involved in revising the manuscript critically for intellectual content. RL was involved in revising the manuscript critically for intellectual content and gave final approval of the version to be published. RA performed *cis*-elements identification and wrote the corresponding part of the manuscript. ML conceived the study, carried out the genome search, the gene and promoter sequences analysis, the phylogenetic analysis, participated in building macroarrays and wrote the manuscript. All authors read and approved the final manuscript.

## Supplementary Material

Additional file 1 **Sucrose and Monosaccharide transporter genes identified in *Vitis vinifera *genome**. Vitis proteome 8× ID, attributed name, chromosomal position, gene length, number of introns and exons, ORF length, protein length, estimated protein molecular weight and pI, GenBank ID and reference are indicates when available. Genes written in italics are partial. Click here for file

Additional file 2 **Sucrose and Monosaccharide transporter genes promoter sequences identified in *Vitis vinifera *genome**. Vitis proteome 8× ID, attributed name, chromosomal position and promoter length are indicated. Click here for file

Additional file 3 ***Cis*-acting elements potentially involved in sugar-regulated transcription identified in the *VvSUC/VvSUT*, *VvHT*, *VvTMT *and *VvPMT *promoter sequences**. Promoter sequence analysis was performed *via *PLACE. *Cis*-element names, sequence motifs, signalling pathways and the number of copies for each element are presented for each promoter. x(xx): number of motif types (total number of identified motifs). *i) *Elements for sugar responsiveness. *ii) *Elements for sugar and hormonal signals perception. Click here for file

Additional file 4 **Oligonucleotide primers used to amplify specific 3'UTR of sugar transporter and reference genes**. Gene name, oligonucleotide sequences and length of the amplified cDNA fragments are indicated. Click here for file
