# Supplementary material for: The Vitis vinifera sugar transporter gene family: phylogenetic overview and macroarray expression profiling
Source: BMC Plant Biol. 2010 Nov 12;10:245. doi: 10.1186/1471-2229-10-245 (PMC3095327; doi:10.1186/1471-2229-10-245)
Supplement: Additional file 1 — Sucrose and Monosaccharide transporter genes identified in Vitis vinifera genome. Vitis proteome 8× ID, attributed name, chromosomal position, gene length, number of introns and exons, ORF length, protein length, estimated protein molecular weight and pI, GenBank ID and reference are indicates when available. Genes written in italics are partial. [file 1471-2229-10-245-S1.PDF]

Vitis Proteome 8X ID	Name	Chr	Position	Gene (bp)	Intron	Exon	ORF (bp)	Prot AA	Prot kDa	Prot pI	GenBank ID	Reference
VvSUC/VvSUT												
GSVIVT00015035001	*VvSUC11/VvSUT1*	18	(-) 6597517 - 6607103	9587	4	5	1506	501	54.0	9.11	HQ323256	[30], [31]
GSVIVT00037013001[b]	*VvSUC12*	1	(+) 2476369 - 2486447	10079	13	14	1818	606	64.9	6.49	HQ323257	[30]
GSVIVT00002302001	*VvSUC27*	Un	(+) 20463408 - 20465802	2395	3	4	1518	505	53.9	9.51	HQ323258	[30]
GSVIVT00002307001	*VvSUT2*	Un	(+) 20520485 - 20522864	2380	3	4	1521	506	54.1	8.14	HQ323259	
VvHT												
GSVIVT00004559001	VvHT1	Un	(+) 97905598 - 97908563	2966	3	4	1560	519	57.2	8.91	HQ323260	[35], [36]
GSVIVT00015239001	*VvHT2*	18	(+) 4309655 - 4315053	5399	3	4	1503	500	54.1	9.34	HQ323261	[35], [36]
GSVIVT00005628001	*VvHT3/VvHT7*	Un	(-) 33666349 - 33670069	3721	3	4	1581	526	57.1	8.68	HQ323262	[36], [37]
GSVIVT00030854001	*VvHT4*	Un	(+) 113352409 - 113355948	3540	2	3	1527	508	55.1	9.58	HQ323263	[37]
GSVIVT00019956001	*VvHT5*	5	(-) 4760129 - 4764785	4657	4	5	1611	536	58.9	8.68	HQ323264	[37]
GSVIVT00009747001	*VvHT8*	Un	(+) 64180804 - 64183768	2965	3	4	1560	519	57.2	8.91	HQ323265	
GSVIVT00038185001	*VvHT9*	14	(+) 11120535 - 11122564	2030	3	4	1569	522	57.8	8.84	HQ323266	
GSVIVT00038183001	*VvHT10*	14	(+) 11128172 - 11130189	2018	3	4	1569	522	57.6	8.69	HQ323267	
GSVIVT00038182001	*VvHT11*	14	(+) 11136404 - 11138838	2435	3	4	1608	535	59.0	9.57	HQ323268	
GSVIVT00019953001	*VvHT12*	5	(+) 4743399 - 4745342	1944	3	4	1521	506	56.1	9.28	HQ323269	
GSVIVT00016689001	*VvHT13*	11	(-) 2727346 - 2729634	2289	3	4	1539	512	56.3	9.06	HQ323270	
GSVIVT00025290001	*VvHT14*	9	(-) 13727720 - 13729869	2150	3	4	1521	506	56.0	8.85	HQ323271	
GSVIVT00028620001	*VvHT15*	13	(+) 12400674 - 12402822	2149	3	4	1521	506	56.0	8.75	HQ323272	
GSVIVT00028621001	*VvHT16*	13	(+) 12411681 - 12413829	2149	3	4	1521	506	56.0	8.85	HQ323273	
GSVIVT00028622001	*VvHT17*	13	(+) 12421262 - 12423413	2152	3	4	1521	506	55.9	9.46	HQ323274	
GSVIVT00028624001	*VvHT18*	13	(+) 12433785 - 12435932	2148	3	4	1521	506	55.9	8.85	HQ323275	
GSVIVT00028634001	*VvHT19*	13	(+) 12502337 - 12504485	2149	3	4	1521	506	55.9	8.64	HQ323276	
GSVIVT00028629001[a]	*VvHT20*	*13*	*(+) 12466333 - 12467430*	*1098*	*2*	*3*	*576*	*191*			*HQ323277*	
GSVIVT00028630001[a]	*VvHT21*	*13*	*(+) 12467740 - 12468481*	*742*	*1*	*2*	*636*	*211*			*HQ323278*	
GSVIVT00028635001[bc]	*VvHT22*	*13*	*(+) 12514643 - 12515322*	*680*	*1*	*1*	*639*	*209*			*HQ323279*	
GSVIVT00028636001[bd]	*VvHT23*	*13*	*(+) 12516175 - 12517100*	*926*	*1*	*2*	*801*	*266*			*HQ323280*	
GSVIVT00013228001[e]	*VvHT24*	*Un*	*(-) 152564138 - 152564842*	*705*	*1*	*2*	*455*	*147*			*HQ323281*	
VvTMT												
GSVIVT00002919001	*VvTMT1/VvHT6*	18	(+) 593142 - 598663	5522	4	5	2223	740	79.4	4.78	HQ323282	[36]
GSVIVT00036283001[b]	*VvTMT2*	3	(-) 2853987 - 2859495	5509	4	5	2220	740	79.2	5.15	HQ323283	
GSVIVT00019321001	*VvTMT3*	7	(-) 13510057 - 13514684	4628	4	5	2229	742	80.1	5.07	HQ323284	
VvPMT												
GSVIVT00010278001	*VvPMT1*	Un	(-) 63357751 - 63360120	2370	1	2	1569	522	55.6	7.51	HQ323285	
GSVIVT00016743001	*VvPMT2*	11	(+) 3153566 - 3155163	1598	1	2	1503	500	53.7	7.59	HQ323286	
GSVIVT00024964001	*VvPMT3*	4	(+) 12877974 - 12879707	1734	1	2	1494	497	53.4	9.28	HQ323287	
GSVIVT00025836001	*VvPMT4*	12	(+) 7679763 - 7681888	2126	1	2	1581	526	56.5	5.73	HQ323288	
GSVIVT00036419001	*VvPMT5*	3	(-) 3940283 - 3942288	2006	1	2	1587	528	57.1	8.67	HQ323289	

Vitis Proteome 8X ID	Name	Chr	Position	Gene (bp)	Intron	Exon	ORF (bp)	Prot AA	Prot kDa	Prot Pi	GenBank ID	Reference
VvERD6-Like												
GSVIVT00006081001		14	(-) 3917392 - 3921442	4051	17	18	1467	488	52.0	5.43	HQ323290	
GSVIVT00006082001		14	(-) 3922070 - 3927376	5307	17	18	1467	488	52.1	5.13	HQ323291	
GSVIVT00006083001[bg]		*14*	*(-) 3941248 - 3943374*	*2127*	*8*	*9*	*720*	*240*			*HQ323292*	
GSVIVT00006084001[b]		14	(-) 3947751 - 3955780	8030	17	18	1464	488	53.1	5.68	HQ323293	
GSVIVT00006086001		14	(-) 3969569 - 3973251	3683	17	18	1428	475	51.4	5.03	HQ323294	
GSVIVT00006087001		14	(-) 3974128 - 3978856	4729	17	18	1434	477	51.3	5.42	HQ323295	
GSVIVT00006088001		14	(-) 3981155 - 3985282	4128	17	18	1470	489	52.3	5.41	HQ323296	
GSVIVT00006090001		14	(-) 3992915 - 3997055	4141	16	17	1452	483	52.3	5.59	HQ323297	
GSVIVT00006094001[f]		*14*	*(-) 4025915 - 4027430*	*1516*	*3*	*4*	*351*	*116*			*HQ323298*	
GSVIVT00006096001		14	(-) 4033585 - 4037683	4099	16	17	1452	483	52.6	5.80	HQ323299	
GSVIVT00006097001[b]		14	(-) 4039757 - 4044440	4684	16	17	1296	431	47.9	5.98	HQ323300	
GSVIVT00006098001		14	(-) 4048971 - 4053915	4945	17	18	1473	490	52.6	5.02	HQ323301	
GSVIVT00006099001		14	(-) 4054745 - 4059458	4714	17	18	1425	474	51.2	7.30	HQ323302	
GSVIVT00006100001		14	(-) 4063818 - 4068605	4788	16	17	1422	473	51.4	7.14	HQ323303	
GSVIVT00014605001		18	(-) 10622720 - 10627729	5010	16	17	1419	472	51.1	8.51	HQ323304	
GSVIVT00019852001		5	(+) 3809382 - 3812501	3120	17	18	1317	438	47.0	8.26	HQ323305	
GSVIVT00019859001		5	(+) 3875707 - 3879134	3428	17	18	1467	488	53.5	6.46	HQ323306	
GSVIVT00019860001		5	(+) 3885752 - 3891653	5902	17	18	1467	488	53.4	6.26	HQ323307	
GSVIVT00023634001		7	(-) 2341086 - 2345987	4902	17	18	1473	490	52.6	5.44	HQ323308	
GSVIVT00024920001		4	(-) 13255030 - 13262040	7011	17	18	1461	486	52.6	8.22	HQ323309	
GSVIVT00035473001 [f]		12	(-) 17377258 - 17379290	2033	9	10	858				HQ323310	
GSVIVT00035474001 [f]		12	(-) 17367461 - 17371484	4024	14	15	933				HQ323311	
VvVGT												
GSVIVT00014041001[bd]	*VvVGT1*	16	(-) 2220975 - 2228079	7105	11	12	1363	453	48.1	7.68	HQ323312	
GSVIVT00024269001	*VvVGT2*	6	(-) 6070317 - 6074777	4461	13	14	1686	561	60.1	7.94	HQ323313	
VvINT												
GSVIVT00001923001	*VvINT1*	5	(-) 20201875 - 20234788	32914	5	6	1500	499	53.3	5.25	HQ323314	
GSVIVT00028332001	*VvINT2*	10	(-) 3525662 - 3529939	4278	5	6	1734	577	63.0	7.97	HQ323315	
GSVIVT00012471001	*VvINT3*	Un	(-) 78691247 - 78698710	7464	5	6	1758	585	63.1	8.27	HQ323316	
VvpGlcT												
GSVIVT00025939001 [f]		12	(-) 6629242 - 6635937	6696	10	11	1320	439	47,8	5.35	HQ323317	
GSVIVT00016716001		11	(-) 2943941 - 2953717	9777	12	13	1467	488	52.5	8.10	HQ323318	
GSVIVT00034389001		9	(+) 3962829 - 3970463	7635	11	12	1488	495	53.4	7.00	HQ323319	
GSVIVT00038247001	*VvpGlT*	14	(-) 9924542 - 9932555	8014	12	13	1560	519	55.1	9.36	HQ323320	[20], [36]

[a]: both ID might represent a single gene; [b]: modified annotation compared to Genoscope annotation
[c]: partial ORF with 5' and 3'-ends not sequenced
[d]: partial ORF with 5'-end not sequenced; [e]: partial ORF with 3'-end not sequenced
[f]: partial ORF with 5'-term not clearly identified; [g]: partial ORF with 3'-end not clearly identified
