# Supplementary material for: The Vitis vinifera sugar transporter gene family: phylogenetic overview and macroarray expression profiling
Source: BMC Plant Biol. 2010 Nov 12;10:245. doi: 10.1186/1471-2229-10-245 (PMC3095327; doi:10.1186/1471-2229-10-245)
Supplement: Additional file 2 — Sucrose and Monosaccharide transporter genes promoter sequences identified in Vitis vinifera genome. Vitis proteome 8× ID, attributed name, chromosomal position and promoter length are indicated. [file 1471-2229-10-245-S2.PDF]

Vitis Proteome 8X ID	Names	Chr	Position	Length
VvSUC/VvSUT				
GSVIVT00015035001	*VvSUC11/VvSUT1*	18	(-) 6609103 - 6607104	1997
GSVIVT00037013001	*VvSUC12*	1	(+) 2474369 - 2476479	2108
GSVIVT00002302001	*VvSUC27*	Un	(+) 20461408 - 20463407	1997
GSVIVT00002307001	*VvSUT2*	Un	(+) 20518485 - 20520484	1997
VvHT				
GSVIVT00004559001	*VvHT1*	Un	(+) 97903598 - 97905752	2152
GSVIVT00015239001	*VvHT2*	18	(+) 4307655 - 4309798	2141
GSVIVT00005628001	*VvHT3/VvHT7*	Un	(-) 33672069 - 33669956	2111
GSVIVT00030854001	*VvHT4*	Un	(+) 113350409 - 113352408	1997
GSVIVT00019956001	*VvHT5*	5	(-) 4766785 - 4764786	1997
GSVIVT00009747001	*VvHT8*	Un	(+) 64178804 - 64180958	2152
GSVIVT00038185001	*VvHT9*	14	(+) 11118535 - 11120534	1997
GSVIVT00038183001	*VvHT10*	14	(+) 11126172 - 11128171	1997
GSVIVT00038182001	*VvHT11*	14	(+) 11134404 - 11136403	1997
GSVIVT00019953001	*VvHT12*	5	(+) 4741399 - 4743398	1997
GSVIVT00016689001	*VvHT13*	11	(-) 2731634 - 2729635	1997
GSVIVT00025290001	*VvHT14*	9	(-) 13731327 - 13729870	1455
GSVIVT00028620001	*VvHT15*	13	(+) 12398674 - 12400673	1997
GSVIVT00028621001	*VvHT16*	13	(+) 12409681 - 12411680	1997
GSVIVT00028622001	*VvHT17*	13	(+) 12419262 - 12421261	1997
GSVIVT00028624001	*VvHT18*	13	(+) 12431785 - 12433784	1997
GSVIVT00028634001	*VvHT19*	13	(+) 12500337 - 12502335	1997
VvTMT				
GSVIVT00002919001	*VvTMT1/VvHT6*	18	(+) 591820 - 593819	1997
GSVIVT00036283001	*VvTMT2*	3	(-) 2861495 - 2859490	1997
GSVIVT00019321001	*VvTMT3*	7	(-) 13516306 - 13514679	1619
VvPMT				
GSVIVT00010278001	*VvPMT1*	Un	(-) 63362120 - 63360121	1997
GSVIVT00016743001	*VvPMT2*	11	(+) 3152940 - 3153565	623
GSVIVT00024964001	*VvPMT3*	4	(+) 12875974 - 12877973	1997
GSVIVT00025836001	*VvPMT4*	12	(+) 7677763 - 7679762	1997
GSVIVT00036419001	*VvPMT5*	3	(-) 3944228 - 3942229	1997
