# Supplementary material for: The Vitis vinifera sugar transporter gene family: phylogenetic overview and macroarray expression profiling
Source: BMC Plant Biol. 2010 Nov 12;10:245. doi: 10.1186/1471-2229-10-245 (PMC3095327; doi:10.1186/1471-2229-10-245)
Supplement: Additional file 3 — Cis-acting elements potentially involved in sugar-regulated transcription identified in the VvSUC/VvSUT, VvHT, VvTMT and VvPMT promoter sequences. Promoter sequence analysis was performed via PLACE. Cis-element names, sequence motifs, signalling pathways and the number of copies for each element are presented for each promoter. x(xx): number of motif types (total number of identified motifs). i) Elements for sugar responsiveness. ii) Elements for sugar and hormonal signals perception. [file 1471-2229-10-245-S3.PDF]

Cis- element name	Sequence	Response	SUC11 8(14)	SUC12 6(18)	SUC27 5(9)	SUT2 8(10)	HT1 9(23)	HT2 6(17)	HT3 6(19)	HT4 5(17)	HT5 7(14)	HT8 9 (22)	HT9 5(10)	HT10 5(10)	HT11 6(14)	HT12 6(14)	HT13 5(16)	HT14 5(12)	HT15 5(12)	HT16 5(12)	HT17 6(12)	HT18 5(13)	HT19 5(13)	TMT1 5(11)	TMT2 5(6)	TMT3 7(14)	PMT1 5(12)	PMT2 3(3)	PMT3 4(6)	PMT4 6(9)	PMT5 7(15)
i) SURE1STPAT21	AATAGAAAA	sucrose induction	2			1	2			2		2			2	2		2	1					1		1					1
i) SURE2STPAT21	AATACTAAT	sucrose, root	1		1																										
i) SUCROSE BOX 3	AAATCA…AA	sucrose induction	1	2	2	3	2	2	3	1	2	2	2	2	3	1	3	1		3	3	2	1		1	1	2		1	1	4
i) CGACGOSAMY3	CGACG	sucrose starvation					1		1		2	1			1																
i) CMSRE1IBSPOA	TGGACGG	sucrose induction						1			1															1					
i) SP8BFIBSP8BIB	TACTATT	sucrose induction	2		1	1		2					3	3				1	1		1	1	1		1						2
i) WBOXHVISO1	TGACT	Sugar, SUSIBA2	2	7	3	1	8	9	5	4	3	8	2	2	4	4	5	4	4	2	2	2	3	2	2	2	6	1	3	2	3
ii) SBOXATRBCS	CACCTCCA	Sugar, ABA, ABI4					2					1			1																
ii) MYBGAHV	TAACAAA	GA, sugar repression	2	3		1																		1	1		1	1		1	1
ii) AMYBOX1	TAACARA	sugar, ABA, GA	2	4		1	1	1				1	2	2		2				1	1			1		2	1	1		2	1
ii) AMYBOX2	TATCCAT	sugar, ABA, GA					2		3		1	2					2				1	3	2								
ii) PYRIMIDINEBOXHVEPB1	TTTTTTCC	GA, ABA, sugar, seeds		1		1	1		1	1	2	1				2	2		1	2						2			1	1	
ii) PYRIMIDINEBOXOSRAMY1A	CCTTTT	Sugar repression, seeds	2	1	2	1	4	2	6	9	3	4	1	1	3	3	4	4	5	4	4	5	6	6	1	5	2		1	2	3

Found only in *VvSUC/VvSUT*

Found in all promoters except *VvHT15* , *VvTMT1* and *VvPMT2*

Found only in some *VvHT* promoters

Found only in *VvHT2* , *VvHT5* , *VvTMT3*

Not found in *VvHT*

Putative GARC complex
