# Supplementary material for: The Vitis vinifera sugar transporter gene family: phylogenetic overview and macroarray expression profiling
Source: BMC Plant Biol. 2010 Nov 12;10:245. doi: 10.1186/1471-2229-10-245 (PMC3095327; doi:10.1186/1471-2229-10-245)
Supplement: Additional file 4 — Oligonucleotide primers used to amplify specific 3'UTR of sugar transporter and reference genes. Gene name, oligonucleotide sequences and length of the amplified cDNA fragments are indicated. [file 1471-2229-10-245-S4.PDF]

Gene	Primers (5' - 3')	Amplicon (pb)
VvSUC11	F : CCA TGG GAT CAA CTT TTT G<br>R : CAT TTT ACC ACC CAT ATT GAT	191
VvSUC12	F : GGA TGC TTT ATT TGG TGG A<br>R : AGC ACA AGG CAT CAA AGC	396
VvSUC27	F : TGT AGT GGG TGC GTT TGC<br>R : GAT GAC CGT GGG CTC TAC A	369
VvSUT2	F : CTG AAT CGA AGA GTT GGA G<br>R : CTT GTC TCA GCA CTT GGC	326
VvHT1	F : TCA ACG ATG GTT CTT ACA GC<br>R : AAC CGA TAG TAT TGT ATT CG	262
VvHT2	F : GCG GTG TTG GTA CTG GTA C<br>R : GGA CTT GAG AAG GAA TTT AGC	371
VvHT3	F : CAC TTG GGT GGA CAG TTC C<br>R : GGA TTA TTT GAA GTT CAC CGA	400
VvHT4	F : ATT CGT GTA TTA TTT GCT GC<br>R : CGG GCA ATT TAC GAT CAT	333
VvHT5	F : GGT GTG GAA GAA GCA TTG G<br>R : GGC ACA AGA ACA CAT ATA C	323
VvHT11	F : GGA AAG GCA ACC CAA AGT G<br>R : CTA TTT ATT GTA ATT TGA ATG C	187
VvHT12	F : AGA TGA GCT AGT GGC ACA AC<br>R : AGA TAT CAT GGT GGA TGG	224
VvHT13	F : GGA CTA CTC TAT TGA AAT CC<br>R : GGA AAT TCT TTG TTT ATA CTG C	139
VvTMT1	F : TGA CTG AAT GCG GGG GAA GC<br>R : GTG TGG ATA TTT TGA TGG TCG	109
VvTMT2	F : TTT CGA TTC AGA TGC TCC<br>R : CAT CAG AGA GCC CCT GAA AG	240
VvTMT3	F : CAT CCT GGA ACT GCC GGG<br>R : CAT CAA AGA ATG CTG AGC	230
VvPMT1	F : AGC GTG CTT ATG CTG AAC CAC<br>R : AGG GCT AGA GAC AAA CTA TAA	332
VvPMT2	F : AAC CCT TAC TTA CCG ATA G<br>R : GGG ATC GAC GGA CAC CTG ATA	308
VvPMT3	F : CGT TTT ATA CTG GTT TAT TG<br>R : AGT GAA GCC GCA TAT GCT	310
VvPMT4	F : AAG TTT CAT TTT ATG AGC TAA<br>R : GCC AGT GAC TGG AAC AGC CAG	243
VvPMT5	F : AGA AGA AGT CAT GTA CTA AC<br>R : TTC ATT CCA TAT TCA GCC CC	222
VvEF1α	F : GAA CTG GGT GCT TGA TAG GC<br>R : AAC CAA AAT ATC CGG AGT AAA AGA	168
VvEF1γ	F : AGC TTT TAC CGC GGG CAA GAG ATA CC<br>R : TTT GGA TAG GTA ACG TAT CAC TTA AAT AAC	318
VvGAPDH	F : CGA CCA TTG TTA CTG CTG T<br>R : GAA ATC CAG GGG CAA AAC	377
VvActin	F : AGC TGG AAA CTG CAA AGA GCA G<br>R : ACA ACG GAA TCT CTC AGC TCC A	95
